# Functional Avoidance Liver 4*π* Stereotactic Body Radiation Therapy Informed by Quantitative Gadoxetic Acid Contrast-Enhanced Magnetic Resonance T1 Mapping

**DOI:** 10.1016/j.ijrobp.2025.09.051

**Published:** 2025-09-27

**Authors:** Joshua Everts, Yang Yang, Qifan Xu, Qihui Lyu, Mary Feng, Jessica Scholey, Michael Ohliger, William C. Chen, Alexandra E. Hotca, Mekhail Anwar, Junzhou Chen, Zhaoyang Fan, Ke Sheng, Wensha Yang

**Affiliations:** a Department of Radiation Oncology, UC San Francisco, San Francisco, California; b Department of Radiology, UC San Francisco, San Francisco, California; c Department of Radiology, University of Southern California, Los Angeles, California

## Abstract

**Purpose:**

Recent studies have identified the T1 reduction rates (k1) from gadoxetic acid–enhanced magnetic resonance (MR) imaging as a biomarker for liver function. In this study, we validate k1 maps as a functional biomarker and develop 4*π* noncoplanar treatment plans using k1 maps to guide the optimization for liver functional avoidance stereotactic body radiation therapy (FA-SBRT).

**Methods and Materials:**

One hundred six patients underwent precontrast and postcontrast T1 mapping MR. Mean k1 values extracted from liver mask excluding gross tumor volume (liver-GTV) were correlated with Child-Pugh and albumin-bilirubin scores. The high-function (HF) liver region was identified and masked using a patient-specific threshold from the Gaussian decomposition of the k1 histogram. Twenty patients were retrospectively planned with coplanar and noncoplanar 4*π*-SBRT with and without functional avoidance. Tumor coverage was maintained at a minimum of 90% planning target volume to receive prescription, and standard organ at risk constraints were set for all plans. Dose metrics included mean dose to the HF liver and HF liver volume receiving 6 Gy, which was shown to impact patient liver function. A paired, 2-tailed *t* test was used to determine the statistical significance.

**Results:**

k1 values were inversely correlated with Child-Pugh and albumin-bilirubin scores. The 4*π* FA-SBRT plans reduced the mean dose to the HF liver volume by 21.8% (from 9.2 Gy to 6.5 Gy) (*P* < .0001) and the volume of HF liver receiving >6 Gy by 39.5% (from 507.5 cm^3^ to 302.2 cm^3^) compared with the 20-beam coplanar geometry (*P* < .0001). All reductions were statistically significant (*P* < .01).

**Conclusions:**

This study validates k1 derived from free-breathing T1 mapping MR as a liver function biomarker in a cancer patient cohort. Gaussian decomposition can threshold the k1 distribution to create patient-specific HF liver masks. The 4*π* FA-SBRT planning guided by k1 maps significantly reduced the mean dose to the HF liver, as well as the volume of HF receiving >6 Gy.

## Introduction

Liver cancer remains one of the most pressing global health challenges, ranking as the third leading cause of death globally, with over 800,000 annual mortalities in 2020.^1^ Among liver cancers, hepatocellular carcinoma (HCC) is the most prevalent and is often associated with underlying chronic liver diseases such as hepatitis B, hepatitis C, or cirrhosis. Unfortunately, treatment options for HCC are usually limited, particularly for patients who are ineligible for standard interventions such as surgery, chemotherapy, or locally ablative therapies like Y90 radioembolization, microwave ablation, or cryotherapy.^2^ For these patients, stereotactic body radiation therapy (SBRT) has emerged as a promising therapeutic alternative, offering precise tumor targeting with manageable toxicity to surrounding healthy tissue.^3–6^

The liver is also a common site for metastatic disease, originating from primary sites such as the colorectum, breast, pancreas, lung, stomach, and neuroendocrine system.^7^ According to the surveillance, epidemiology, and end result database, approximately 5% of all cancer patients present with synchronous liver metastases at the time of diagnosis.^8^ Treatment strategies for liver metastases often mirror those for HCC, with SBRT proving to be an effective and well-tolerated option in this population. Evidence supports its role in providing local control and symptom relief, even in cases where other therapies are unsuitable.^9^

Despite its established efficacy in carefully selected patients, SBRT for liver cancer is associated with significant challenges, particularly in the context of underlying hepatic dysfunction or fibrosis. The potential for treatment-related toxicity underscores the necessity for careful patient selection and the development of innovative strategies to optimize therapeutic outcomes. One of the most critical complications of liver SBRT is radiation-induced liver disease, affecting 20% to 30% of patients.^10^ This condition is particularly prevalent in patients with underlying liver fibrosis or reduced functional liver volume, such as those with Child-Pugh (CP) class B or C liver function. The severe sequelae of radiation-induced liver disease, including irreversible hepatic failure, reactivation of hepatitis B, and mortality, significantly constrain therapeutic options for these high-risk cohorts.^11^ Many patients in these higher-risk groups, specifically those with liver fibrosis CPB or CPC, experience disproportionately higher toxicity rates compared to CPA patients, necessitating a careful evaluation of the risk-to-benefit ratio, further limiting their treatment options.^12^

Additionally, liver metastases are characterized by high recurrence rates, frequently requiring repeated SBRT courses for disease control.^13,14^ With the increasing survival of cancer patients, the paradigm in liver SBRT has evolved from achieving tumor ablation to prioritizing the preservation of functional hepatic subvolumes. This approach aims to mitigate radiation exposure to critical liver regions, thereby preserving hepatic function and enabling subsequent therapeutic interventions.^15,16^

Functional avoidance (FA)-SBRT (FA-SBRT) represents an advancement in radiation oncology, offering a personalized approach to liver cancer treatment. This technique is designed to selectively spare high-functioning liver subvolumes while ensuring the delivery of optimal therapeutic doses to tumors. By minimizing radiation exposure to healthy and essential liver tissue, FA-SBRT aims to preserve overall liver function, enhance patient quality of life, and enable future treatment opportunities. Studies supporting this approach highlight its potential to revolutionize SBRT for liver cancer and metastases.^17–19^ The cornerstone of FA-SBRT is the integration of reliable voxel-wise quantitative imaging biomarkers to assess and map baseline liver function. Various imaging technologies, such as dual-energy computed tomography (CT), single-photon emission CT (SPECT), positron emission tomography, and magnetic resonance imaging (MRI), have all been used to differentiate highly functional liver volumes from poorly functional ones.^20–22^ Efforts to implement functional liver-sparing strategies using commercial treatment planning systems, such as intensity modulated radiation therapy (IMRT) and volumetric-modulated arc therapy (VMAT), have shown promising results in preliminary studies.^20,23–25^ However, despite these successes, the clinical adoption of FA-SBRT remains limited. A recent comprehensive review concluded that insufficient clinical data exist to definitively confirm the efficacy of these approaches, primarily due to significant challenges in imaging precision, the lack of standardized metrics for liver function quantification, and limitations in the capabilities of existing planning systems.^26^

Gadoxetic acid (Gd-EOB-DTPA), a liver-specific contrast agent combined with quantitative magnetic resonance (MR) T1 mapping, has emerged as a promising biomarker for assessing liver function, offering significant advantages over traditional imaging modalities. Unlike Tc-99m SPECT, which involves ionizing radiation, Gd-EOB-DTPA contrast-enhanced MRI offers superior spatial resolution without exposing patients to radiation risks, making it an attractive alternative for functional liver evaluation.^27–29^ Among the various metrics, the T1 reduction rate (k1) has been identified as a particularly robust indicator of liver function. A key advantage of k1 is its invariance to magnetic field strength, ensuring consistent and reliable measurements across different MRI systems and settings.^30–33^ These attributes position Gd-EOB-DTPA contrast-enhanced imaging as a highly effective tool for mapping liver function in clinical and research settings.

However, many previous studies, leveraging Gd-EOB-DTPA MRI, have relied on breath-hold (BH) or triggered imaging sequences. These methods pose challenges for patients who struggle with breath-holding, particularly those with compromised health or irregular breathing patterns. Furthermore, triggered sequences can result in variability in image acquisition times, impacting the reliability and reproducibility of functional maps. To address these limitations, this study employs the magnetization-prepared golden-angle radial sparse parallel (MP-GRASP) free-breathing sequence, which has been validated for producing reliable quantitative T1 maps in both phantom studies and healthy volunteers.^34^

Although functional imaging has advanced significantly, integrating these maps into SBRT treatment planning presents challenges, especially with traditional coplanar radiation planning techniques, as liver functional maps introduce additional complexity to the optimization process. Noncoplanar 4*π* radiation therapy offers a unique opportunity to exploit the 3-dimensional spatial information from functional maps, outperforming coplanar VMAT in liver SBRT.^35^ Previous studies have demonstrated that manual noncoplanar VMAT techniques achieve only modest improvements,^36^ and data-driven analyses confirm the superiority of 4*π* optimization in sparing functional liver regions.^37^ Current commercial VMAT/IMRT treatment planning systems fail to fully leverage these advancements, limiting potential gains in liver function preservation.

This study aims to bridge the gap between advanced liver function mapping and optimized treatment delivery by combining MP-GRASP–derived T1 mapping with 4*π* planning techniques. By replanning treatment for a cohort of patients, we explore the feasibility and potential therapeutic advantages of functional liver-sparing SBRT using these cutting-edge approaches.

## Methods and Materials

### Patient selection

The study protocol was approved by the UCSF institutional review board (IRB#21-33858). Patients presenting to the radiation oncology department at our institution with a diagnosis of liver cancer or cholangiocarcinoma between May 2023 and April 2025 were evaluated for inclusion. A total of 106 patients who underwent radiation therapy to the liver region and same-day CT and 3 Tesla MR simulation as part of their treatment planning were included. Clinical records and data were anonymized and deidentified before analysis. Twenty patients from this group were selected for the replanning study based on their likelihood of benefiting from liver FA radiation therapy. Replanning selection criteria include the presence of liver cirrhosis (CPA or higher), large tumor (planning target volume [PTV] >100 cm^3^), or previous history of liver-directed therapy (LDT). A summary of patient demographics is provided in Table 1.

**Fig. 1.**
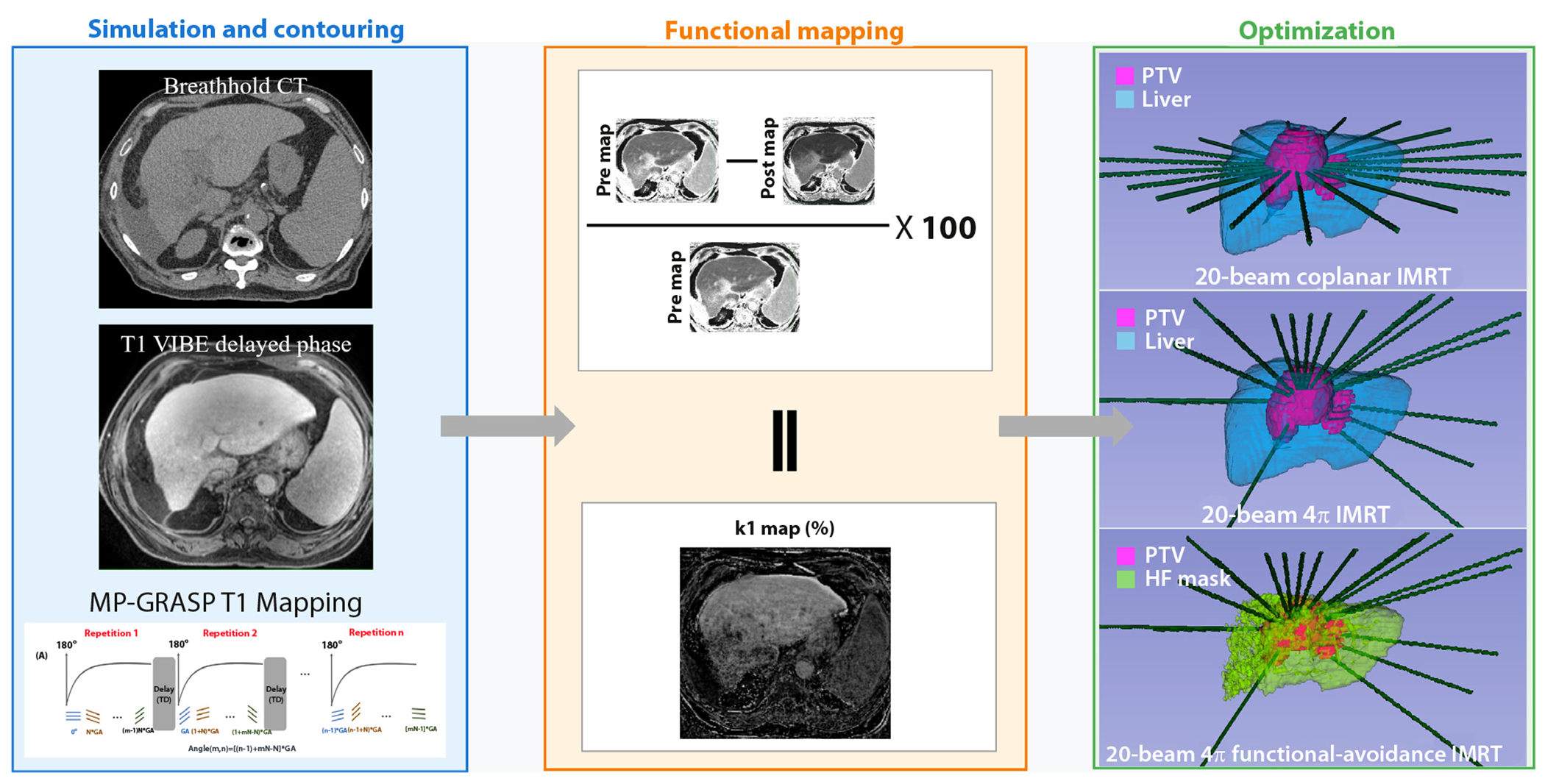
Illustration of the study workflow. Abbreviations: CT = computed tomography; HF = high-function; IMRT = intensity modulated radiation therapy; MP-GRASP = magnetization-prepared golden-angle radial sparse parallel; PTV = planning target volume; VIBE = volume interpolated breath-hold examination.

Figure 1 illustrates the workflow of the study, which is described in detail below.

### Image acquisition

Patients were scanned with identical positioning and immobilization for standard-of-care radiation therapy. Typically, the patients were supine, immobilized using a vacuum body bag, and scanned with arms up. The clinical CT protocol included BH precontrast and postcontrast venous phase scans for target delineation, followed immediately by 4-dimensional CT for motion assessment. The clinical MR protocol included BH precontrast T1-weighted spoiled gradient echo with fat saturation (volume interpolated breathhold examination), T2-weighted balanced steady state free precession line acquisition with undersampling (BLADE), dynamic contrast-enhanced T1-weighted spoiled gradient echo, and echo-planar diffusion-weighted imaging. A research MP-GRASP scan was acquired pre-Gd-EOB-DTPA contrast injection and 20 minutes post-Gd-EOB-DTPA contrast injection, based on patient weight per standard-of-care.^38^ Both precontrast and postcontrast scans were obtained within the same study session to limit interscan motion. The acquired k-space data were saved and reconstructed on a secure research server.

The detailed MP-GRASP sequence, which provides quantitative liver mapping, was previously published.^34^ In brief, an adiabatic 180° inversion recovery pulse is applied before a series of radial stack-of-stars acquisitions. Subsequent repetitions rotate the acquisition stack by golden angles (GA = 111.25°) to enable efficient and uniform k-space coverage. The total imaging time was 3.35 minutes, with a spatial resolution of 1.4 × 1.4 × 5 mm^3^. Appendix E1 provides the details of the sequence implementation. MP-GRASP interscan repeatability was tested in both phantoms and human volunteers in the previous publication, achieving interscan correlations of R^2^ = 1.0 and R^2^ = 0.82, respectively.^34^ Further test-retest scans were performed for 3 patients on our radiation oncology dedicated scanner. MP-GRASP was repeated before contrast injection and during the same study session. An intrasession liver T1 variation of <1% was observed.

### Image reconstruction and processing

Clinical CT and MR images were reconstructed according to vendor-provided protocols. For the research MP-GRASP scans, an in-house postimaging processing pipeline was built in MATLAB (MathWorks). Image reconstruction is performed slice by slice, allowing for the sorting and synchronization of radial k-space samples. Fast subspace-based low-rank and sparsity-constrained GRASP reconstruction (GRASP-Pro: GRASP MRI with imProved performance)^39^ is performed for dynamic image reconstruction of multiple inversion times. Pixel-by-pixel fitting based on the 3-parameter model^40^ is performed on the multiple inversion time dynamic images to calculate the T1 map.

T1_pre_ and T1_post_ maps were clipped to the previously reported range^34^ of (0, 2000) ms to remove unphysical values. T1_pre_ and T1_post_ maps were then rigidly coregistered before image subtraction to correct bulk motion. T1 reduction rate maps were generated via voxel-wise operations using the equation: $k1=\frac{T1pre-T1post}{T1pre}\times 100$. Resultant k1 values were clipped to the range (0, 100) to remove unphysical negative k1 values, which occurred in lung and bowel pockets due to subtraction of thermal and imaging noise. Precontrast T1, postcontrast T1, and k1 raw image arrays were attached with patient-specific digital imaging and communications in medicine (DICOM) image headers compatible with the clinical MIM (MIM Software Inc) system for functional liver segmentation.

### Functional liver definition

Functional liver metrics were calculated using gross target volume (GTV) and liver contours. The k1 map was rigidly registered to the SBRT planning CT scan with each patient’s associated target, liver, and organs at risk (OARs). The liver contour was used to assess registration accuracy, which was manually modified as needed. The liver contour was contracted by 5 mm in the L-R and A-P planes and by 2.5 mm in the S-I plane to further minimize the effects of patient movement and misregistration on the k1 value. The liver mask, excluding GTV (liver-GTV), was then used to derive the mean and standard deviation of the k1 values.

Functional threshold values indicating the high-function (HF) liver cut-off were generated from the liver-GTV maps. The liver-GTV voxel histogram was created using integer-valued bins and approximated using a Gaussian mixture model (GMM). The GMM automatically selected the distribution parameters for bi-Gaussian distributions and was run for 5 iterations to account for estimation uncertainty before selecting the model with the highest log-likelihood. The left intersection between the 2 Gaussian distributions was taken as the functional threshold for each patient, as it represents the point at which a voxel has a greater than 50% chance of having a higher function. Some patients with non-bimodal distributions benefited from a tri-Gaussian GMM, which used a third, smaller Gaussian distribution to account for the remaining variation. For these patients, the intersection between the 2 largest Gaussians was used. Functional threshold k1 values were used to generate an HF liver mask, including all voxels exceeding the threshold. The contour smoothing tool in MIM was applied to enhance the interpretability of contours. The HF mask was then saved and resampled to the patient’s existing structure for treatment planning optimization.

### Treatment planning

#### Treatment planning system

An in-house treatment planning system was developed and previously reported,^41^ with a manuscript under review. Dose calculation was performed using a variant of the collapsed-cone convolution/superposition algorithm, with the resulting data subsequently sparsified on the GPU using tools from the cuSPARSE library. In-house optimization software, written in CUDA and employing the fast iterative shrinkage thresholding algorithm,^42^ was used for beam angle selection and fluence map optimization to minimize the objective function. Dose calculation and optimization steps were performed on an NVIDIA A6000 GPU with 48 GB of memory.

The 4*π* planning method optimizes beam angles by minimizing an objective function that balances 3 terms: dose fidelity, fluence map smoothness, and group sparsity. The dose fidelity term ensures that the resulting dose closely matches the prescribed dose, whereas the fluence map smoothness term promotes a smoother fluence map for improved beam deliverability. The group sparsity term controls the number of activated beams from the candidate list. Following beam selection, fluence map optimization is performed using the dose fidelity and smoothness terms to refine the fluence maps of the selected beams. The 4*π* non-coplanar geometry enables beams to originate from any direction within the 4*π* solid angle, excluding those that intersect the CT cut-off planes or pose a risk of machine-patient collision. Others in our group have previously proposed and validated methods for more sophisticated collision avoidance using the same 4*π* geometry, which would be employed in a prospective treatment plan.^43,44^

#### Contours and dose constraints

Before optimization, all clinical structures, including the HF contour, were converted to binary masks. PTV overlap was removed from structures near the tumor, with a 2-voxel isotropic dilation around the PTV. Dose to clinical OARs and the HF mask were penalized via an L2-norm term. OAR constraints specified in AAPM TG-101 were followed.^45^ A minimum of 95% PTV received the prescription dose (V100 >95%) for various planning geometries. Table E1 describes PTV and OAR goals in detail.

### General planning strategy

Plans were generated with 20 intensity modulated beams using identical beam parameters for each plan type. A 50 Gy initial prescription dose in 5 fractions was applied for each plan type, with an initial penalty of weight 2 for dose >12 Gy placed on the liver-GTV for non-FA plans. OAR dose was assessed against clinical constraints. OAR penalties were iteratively increased for structures that did not meet constraints until satisfied. If a deliverable plan was not found, the prescription dose was lowered in 5 Gy increments until OAR constraints were met. All plans that could meet a 25 Gy prescription dose at a minimum of 90% PTV coverage were included in the final comparison.

### Coplanar planning

Coplanar plans were generated using 20 uniformly spaced coplanar beams to mimic arc planning typically adopted in the clinical plans, while allowing more modulation at fixed gantry angles. Structure weights and PTV prescription dose were individually reoptimized for each coplanar plan during fluence map optimization. Liver-GTV penalties were maintained at the level set in the 4*π* plan. HF liver penalties were not employed here to maintain direct comparison with non-FA 4*π* plans.

### Non-FA 4*π* plans

4*π* plans were generated using 20 beams selected from a safely deliverable subset of possible beam angles. Planning strategy and OAR weights were held identical to the coplanar plans, with OAR weights iteratively adjusted as necessary to meet PTV and OAR targets. The L2 liver-GTV penalty was held identical to the coplanar plan; the HF liver penalty was set to 0 for this plan.

### FA 4*π* plans

FA plans employed the prescription dose and OAR weights optimized in the non-FA, with the only modification being that the previous liver-GTV penalty was replaced with an identical penalty on the HF liver mask. Plans were reoptimized, and OAR penalties were minimally adjusted to ensure similar PTV coverage and total liver sparing. In some cases, a small liver-GTV penalty was added to ensure that the dose to the whole liver volume remained no higher than that of the non-FA 4*π* plan.

### Evaluation metrics are described below.

#### Correlation of k1 with the global liver function biomarker

Patients were categorized into 3 groups: no cirrhosis, CPA, and CPB/C. The mean k1 values generated from the liver-GTV volumes were correlated with each patient’s CP score, a clinical indicator of liver fibrosis. In addition, patients’ albumin-bilirubin (ALBI) grades were calculated from the blood test results within 1 month of the MRI study. Groupwise comparisons were made with a 2-tailed heteroscedasticity-corrected Student *t*test. A regression analysis was performed in Python with the StatsModels package^46^ using an ordinary least squares model. Mean k1 values were correlated with the CP score. Patient age, sex, cancer type, and the presence of prior LDT, which was encoded as a binary variable, were included as covariates in the model.

#### Plan evaluation metrics and statistical comparison

Following the optimization of the coplanar plans, the 4*π* plans were rescaled to match the coplanar prescription dose, allowing for a comparison between the 2 beam geometries. Then, the mean dose, volume receiving more than 6 Gy (V6), and volume receiving more than 12 Gy (V12) were calculated for both the HF liver and the total liver-GTV volume. Statistical comparisons across the mean HF liver dose and HF liver receiving >6 Gy were made using paired, 2-tailed, heteroscedasticity-corrected Student *t*tests.

## Results

### Patient demographics

Patients selected for the planning study were previously treated with clinical liver SBRT plans at our institution. Patient characteristics are summarized in Table 1. Patients with liver metastases formed the largest subgroup (54%), whereas HCC patients comprised 30% of the patients, and intrahepatic cholangiocarcinoma or cholangiocarcinoma patients comprised 16% of the total 106 patients analyzed. Out of 20 patients in the planning study, 8 had HCC, 5 had intrahepatic cholangiocarcinoma or cholangiocarcinoma, and 7 showed metastases.

### k1 map and HF liver definition

**Fig. 2.**
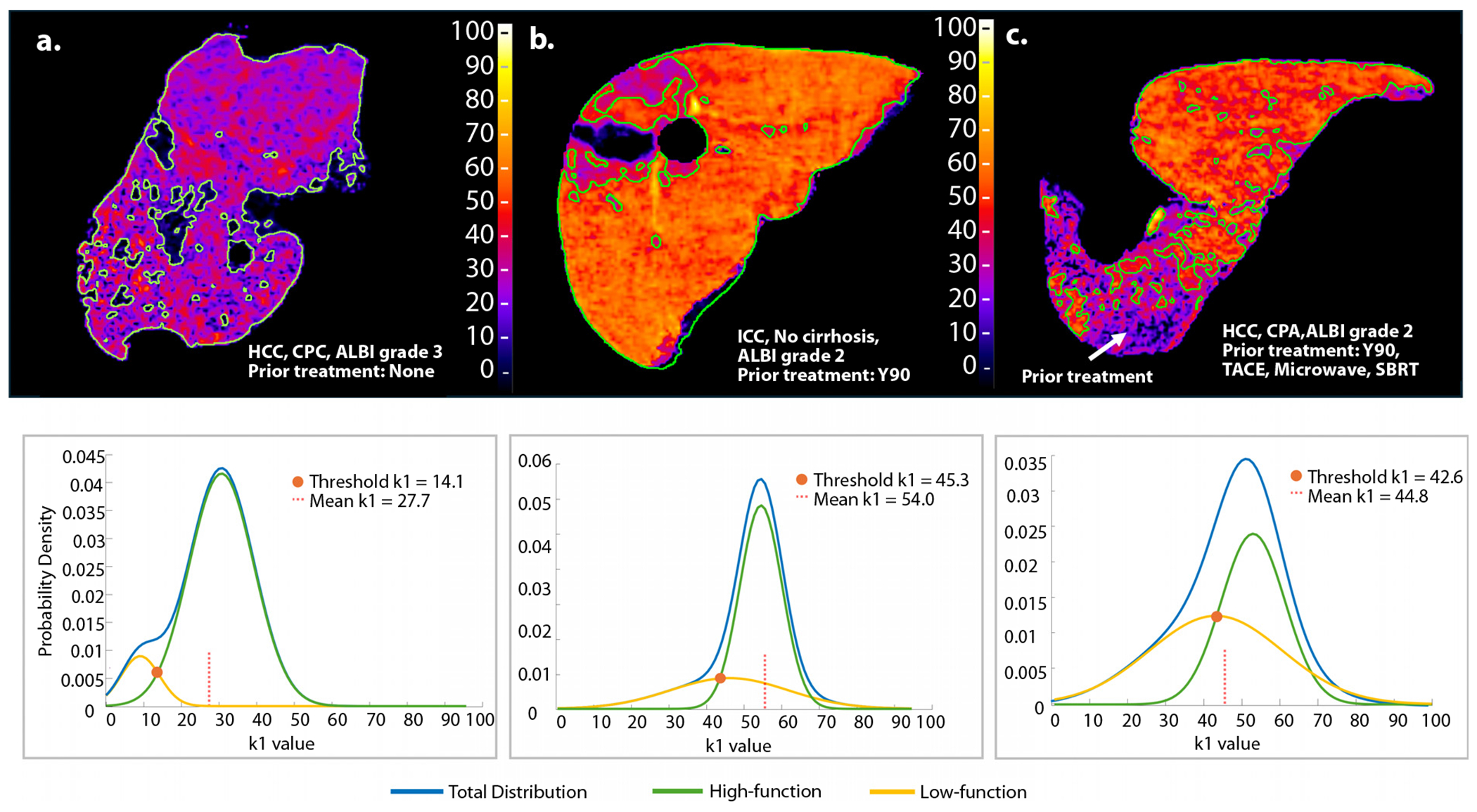
k1 maps, histograms, and high-function liver mask thresholds for 3 typical liver cancer patients. (a) One hepatocellular carcinoma (HCC) patient with Child-Pugh (CP)C cirrhosis score, albumin-bilirubin (ALBI) grade 3, and no prior liver-directed therapy. The patient presents with uniformly low k1 values. (b) One intrahepatic cholangiocarcinoma (ICC) patient without cirrhosis, ALBI grade 2, and prior Y90 treatment. The patient presents with uniformly high k1 values. (c) Another HCC patient with a CPA cirrhosis score, ALBI grade 2, and multiple rounds of liver-directed therapies. The patient has patches of high and low k1 regions, indicating the differential distribution of liver function. Abbreviation: SBRT = stereotactic body radiation therapy.

Figure 2 illustrates how the k1 map distinguishes between less functional (hypointense) and higher functional (hyperintense) regions, with liver intensity proportional to contrast uptake and functional hepatocyte density. The ability of k1 to differentiate voxel-wise liver function is demonstrated. As shown in Figure 2a, global liver function is greatly lowered due to the patient’s high fibrosis score (CPC, ALBI grade 3), which shows up as hypointense compared to the normal liver shown in Figure 2b and CPA fibrosis shown in Figure 2c. As shown in Figure 2b, the patient has normal liver function and displays a relatively homogenous k1 map. Figure 2c shows a region previously treated, which appears hypointense in the right posterior segment of the liver and displays a heterogeneous distribution consistent with a higher fibrosis score (CPA, ALBI grade 2). Together, these results provide qualitative evidence that suggests the potential of the k1 map to localize and quantify less functional liver tissue in patients who have liver fibrosis or have already received previous LDT.

For each patient in Figure 2, the corresponding liver-GTV k1 histogram and bi-Gaussian decompositions are shown. The resulting Gaussian intersection represents an underlying “higher function” cut-off value for the patient. Voxels above the threshold have a greater than 50% chance of belonging to the HF distribution. The HF liver masks are overlaid onto the k1 maps.

### Correlating k1 values with overall liver function

**Fig. 3.**
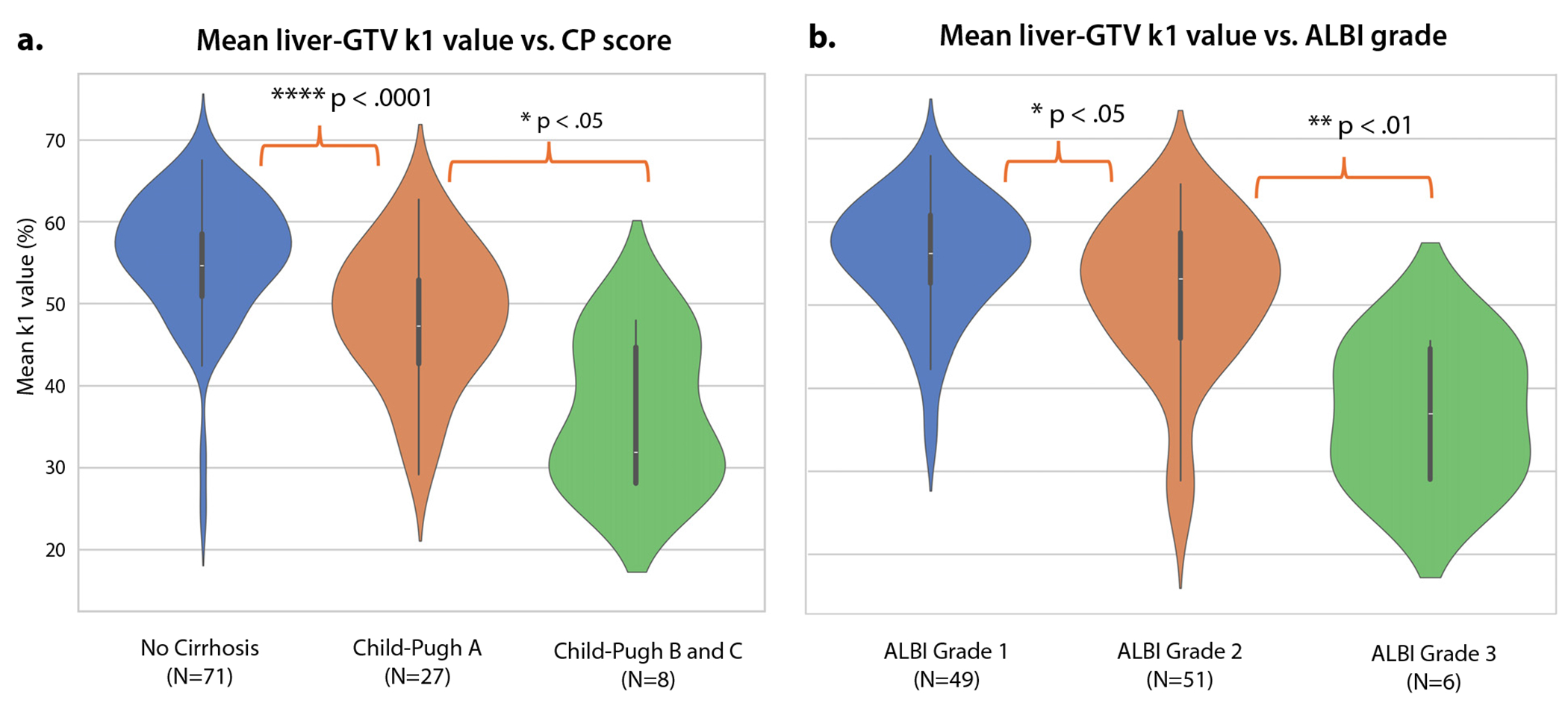
Violin plots of mean k1 value derived from the liver mask, excluding GTV (liver-GTV) of each patient, categorized by Child-Pugh (CP) score (a) and albumin-bilirubin (ALBI) grade (b). Lower mean liver-GTV k1 values are associated with higher CP scores, high ALBI grades, and, therefore, lower liver function. Abbreviation: GTV = gross target volume.

The relationship between the mean liver-GTV k1 value and the CP score was found to be statistically significant (*P* < .0001, *b* = −6.6) with an R^2^ of 0.327. CP score maintained a statistically significant relationship with mean k1 value after controlling for age, sex, cancer type, and presence of prior LDT. Out of these covariates, only pancreatic patients with liver metastases were found to be significant (*P* < .05, *b* = −11.2). Similarly, the relationship between k1 and ALBI was statistically significant (*P* < .0001, *b* = −6.5) with an R^2^ of 0.276, which was maintained after controlling for the same covariates. In this comparison, the presence of HCC (*P* < .05, *b* = −7.1) and pancreatic cancer with liver metastases (*P* < .05, *b* = −7.8) were found to be statistically significant. The distributions of patient k1 values for each CP category are shown in Figure 3a. Patients in the no cirrhosis category had a higher mean k1 value than the CPA category (*P* < .0001), whereas the CPA category had higher mean k1 values than the CPB/C category (*P* < .05). Mean k1 values were also correlated with ALBI grades for each patient. The distributions of the k1 in each ALBI grade category are shown in Figure 3b. The mean liver-GTV k1 values significantly differentiated ALBI grade 1 and grade 2, as well as ALBI grade 2 and grade 3, with *P* < .05 and *P* < .01, respectively.

### Treatment planning results

Treatment plan optimization time using in-house software ranged from 30 seconds to 300 seconds per optimization, depending on PTV size, chosen OAR weights, and OAR geometry. Dose calculation time ranged from 30 seconds to 100 seconds.

**Fig. 4.**
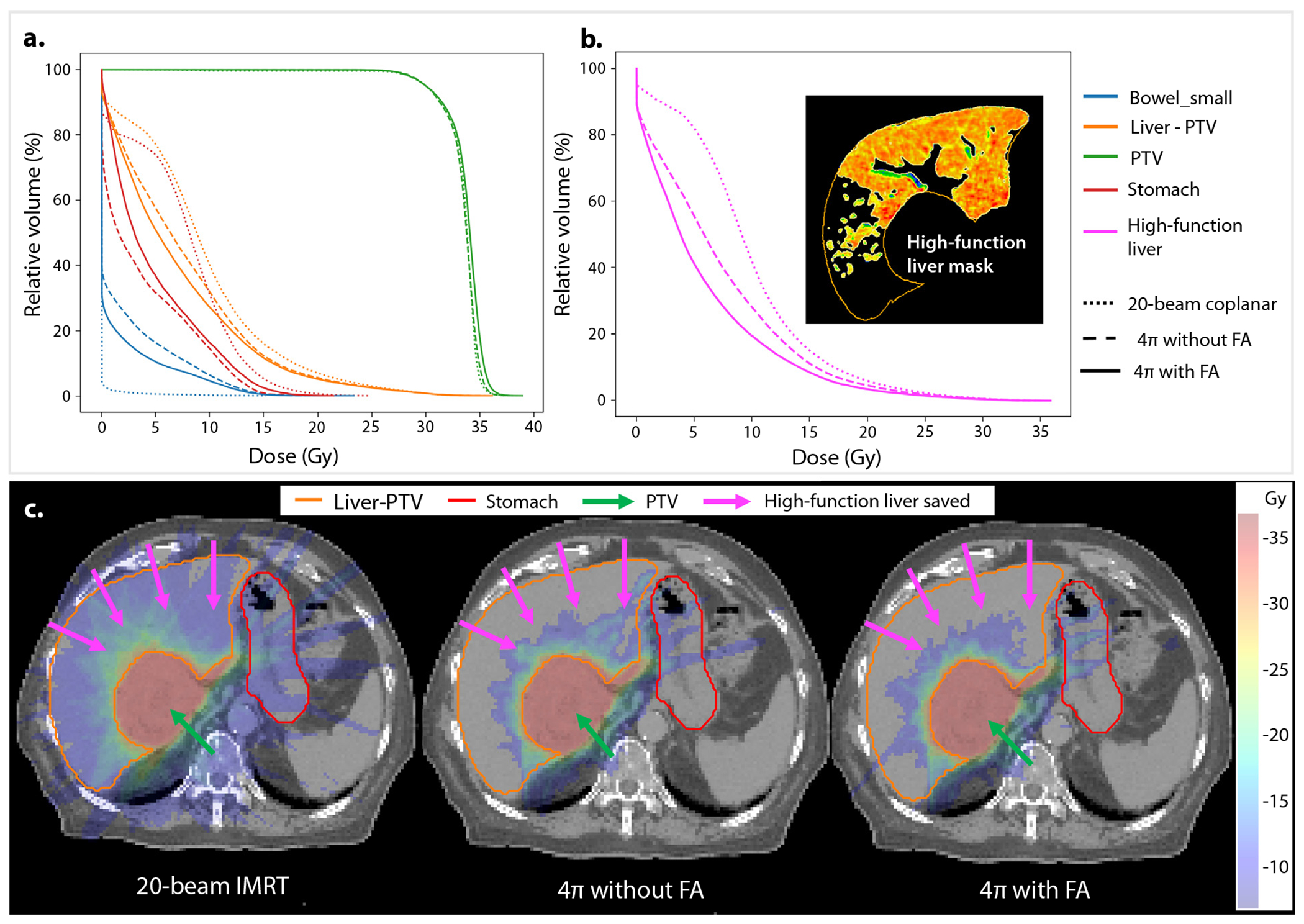
(a) Dose-volume histogram (DVH) of organs at risk and planning target volume (PTV) for an example patient from different plans, (b) high-function (HF) liver mask for the same patient, and DVH of the HF liver volume, (c) dose color washes for the same patient’s plan using 20-beam coplanar, 4*π*-without-FA, and 4*π*-with-FA. Abbreviations: FA = functional avoidance; IMRT = intensity modulated radiation therapy.

Figure 4 demonstrates the potential of the 4*π* noncoplanar geometry and functional mask to spare functional liver dose while maintaining OAR constraints. Dose-volume histograms for OARs and PTV are shown in Figure 4a. Both 4*π* with and without FA plans reduce critical OAR doses compared to the coplanar plan. The dose reduction in the HF liver volume is highlighted by the dose-volume histogram in Figure 4b, showing that 4*π*-with-FA further reduces the dose to the HF liver region. The dose color washes for this patient are also shown in Figure 4c, visually demonstrating the sparing of the HF liver zone by the 4*π*-with-FA plan.

**Fig. 5.**
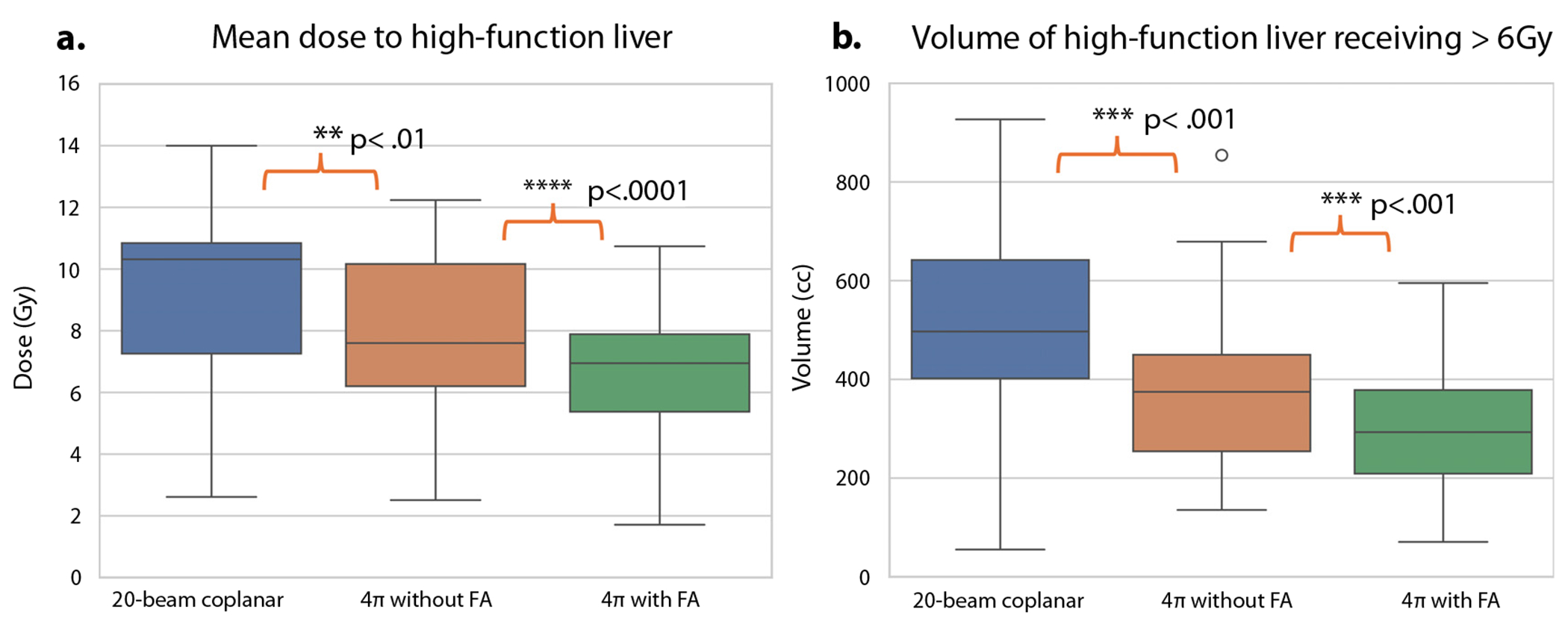
Comparison of mean dose to high-function (HF) liver (a) and volume of HF liver receiving >6 Gy (b) across coplanar, 4*π*-without-FA, and 4*π*-with-FA plans. Mean dose to the HF liver and volume of the HF liver receiving >6 Gy are lowered after applying a 4*π* geometry and further lowered with functional avoidance (FA). Boxplot defined: The central line of each box represents the median; upper and lower box lines represent the third and first quartiles of the distribution. Upper and lower whiskers represent the maximum and minimum of the distribution, barring outliers. Points outside the whiskers are considered outliers.

Figure 5 summarizes the final dose metrics across 20 patients who met the minimal SBRT prescription threshold of 25 Gy for both coplanar and noncoplanar plans. The 4*π* FA-SBRT plans reduced the mean dose to the HF liver volume by 21.8% (from 9.2 Gy to 6.5 Gy) (*P* < .0001) and the volume of HF liver receiving >6 Gy by 39.5% (from 507.5 cc to 302.2 cc) compared with the 20-beam coplanar geometry (*P* < .0001). Final deliverable prescription dose averaged 43.5 Gy with a minimum PTV coverage of 90%.

## Discussion

In this study, we further explored the feasibility of a free-breathing Gd-EOB-DTPA contrast-enhanced T1 mapping as a biomarker for liver function and developed an FA radiation therapy strategy guided by the resulting k1 maps. We found that the mean liver-GTV k1 value was a statistically significant indicator of liver fibrosis level in our cancer patient cohort. Integrating the functional information into noncoplanar 4*π* radiation therapy resulted in a significant dose reduction to the HF liver without compromising OAR constraints or PTV coverage. Coplanar IMRT plans also resulted in significantly inferior tumor dose coverage compared to the 4*π* noncoplanar plans, illustrating the advantage of noncoplanar beams to spare OARs and protect functional liver tissues while maintaining or potentially boosting tumor dose.

Existing work on liver function avoidance radiation therapy has primarily focused on SPECT/CT imaging, yet the low resolution of SPECT and resulting ionizing radiation exposure limit its clinical use.^17,20,24^ In this study, we thus sought to integrate Gd-EOB-DTPA contrast-enhanced MR k1 maps, which have been identified as a robust biomarker for liver function, into a radiation therapy workflow. However, most prior validation of k1 maps has either employed BH sequences, which are limited by patient tolerance, or focused on a healthier, cancer-free patient population.^27,29,47^ In this study, we confirmed the efficacy of free-breathing k1 maps in a liver cancer patient population, which included patients with both primary liver cancers and liver metastases. We found that the free-breathing k1 maps significantly differentiated between CP cirrhosis levels, confirming previous findings from BH sequences. The results suggest that the free-breathing k1 mapping sequence can be integrated into a clinical workflow without affecting liver function quantitation.

In addition to the significant correlations between the CP/ALBI scores and mean k1 value, our analysis revealed substantial interpatient variability in k1 maps, even among patients within the same CP or ALBI category. This variability suggests that the k1 value may be sensitive to voxel-level information that is not captured by the global CP or ALBI score, highlighting the potential for k1 to provide more nuanced insights into liver function. Notably, we also observed a qualitative association between prior LDT and lower k1 values, indicating that k1 may be capable of distinguishing between regions of liver tissue that have been damaged by prior treatment and those that remain highly functional. This finding has important implications for the use of k1 as a biomarker for liver function and its potential application in guiding SBRT optimization and treatment decisions. It shows the advantages of a high-resolution, spatial map of liver function over a global metric like CP or ALBI scores. This finding also mirrors that of Wei et al,^48^ who modeled a fully quantitative contrast uptake rate with dynamic contrast-enhanced (DCE) MRI and found a linear dependence with absorbed dose after SBRT.^38^ Although further work is needed to assess the relationship between the k1 described in our study and absorbed dose, this may suggest that k1 is able to assess similar underlying physiology to DCE MRI, while maintaining greater clinical applicability, as DCE MRI, like all fully quantitative measures, is limited by model parameter choices, long scan times, breathing artifacts, and scanner-dependent thresholds.^28^

Relatively little research has been devoted to defining a threshold for “high-function liver” given a k1 map. Although studies have found that the k1 value of normal livers typically ranges from 60% to 70%,^31,47^ this finding may not translate to a cancer patient population affected by chemotherapy-induced pseudo-cirrhosis or systemic inflammation. Our analysis revealed that a patient-specific threshold, tailored to the individual’s k1 distribution, was the most effective approach for identifying suitable liver tissue for FA-SBRT. This approach considers the spread of k1 values, rather than relying on a single global mean value, which can be influenced by factors such as pseudo-cirrhosis caused by chemotherapy or prior LDT. This custom thresholding strategy is analogous to the approach used in Tc99m-SPECT imaging, where the specific uptake value (SUV) is normalized to the patient’s spleen, and multiple subvolumes are created to spare areas with higher SUV values. By considering the SUV distribution, this method ensures that a wider range of patients can benefit from FA-SBRT, rather than being limited by an absolute global threshold. Our patient-specific approach offers several advantages, including the ability to account for individual variations in liver function and the potential for pseudo-cirrhosis caused by prior treatments. By considering the k1 distribution, we can identify suitable liver tissue for FA-SBRT and ensure that patients receive the most effective treatment possible.

Other studies have attempted to create an FA strategy for liver SBRT using clinical treatment planning systems,^20,22,24–26,49^ which have found small but statistically significant reductions in the volume of liver receiving high dose and the mean functional liver dose, with improvements on the order of 5% to 10%. Although the functional imaging and sparing schemes differed between studies, a 2022 meta-analysis found insufficient evidence that existing FA-SBRT strategies yielded significant dose or volume reductions to the high-functioning liver and concluded that further research was necessary to validate their clinical application.^26^ We believe that an inherent limitation of each study was the use of coplanar arc and beam geometry, which could limit sparing options for the high-functioning liver. In support of this, our FA 4*π* plan demonstrated a reduction in dose to the HF liver by 17% and a reduction in the volume of HF liver receiving >6 Gy by 27%, compared to the 4*π* without FA plans. These reductions compare favorably to coplanar functional sparing plans described above, and come at no cost to total liver dose, which is also reduced. These findings collectively suggest that the 4*π* geometry offers a unique advantage in delivering liver-sparing treatments when combined with functional liver k1 mapping. By leveraging the noncoplanar geometry, clinicians can potentially achieve higher doses to the tumor while minimizing exposure to surrounding healthy tissues, ultimately improving patient outcomes.

Although our study holds promise for achieving significant functional liver sparing in a clinical setting, there are several limitations that must be acknowledged. Firstly, the relatively small number of CPB or CPC patients included in our planning study limits the generalizability of our results to more advanced liver disease cases. Secondly, our current approach to liver sparing is based on a simple binary threshold, which does not fully capture the complexity of liver function distribution or account for factors such as inflammation-mediated liver function decline. Future studies should aim to develop more sophisticated models that can better reflect the nuances of liver function and disease progression. Finally, our treatment planning optimization was performed using in-house dose calculation and beam orientation software, which is not yet approved for clinical use, although we have demonstrated in a prospective clinical trial for glioblastoma that the optimized fluence maps can be imported to a commercially available treatment planning system (Eclipse, Varian, A Siemens Healthineers Company) to allow clinical delivery.^50^ A prospective trial is needed to demonstrate the feasibility of delivering 4*π* treatment on a body site. To advance functional T1 mapping for liver FA-SBRT in the clinic, future work should focus on validating and optimizing methods using commercial software and collaborating with clinical experts. This will enable the development of more effective and personalized functional liver-sparing treatments for patients undergoing SBRT.

## Conclusions

This study shows the feasibility of k1 derived from free-breathing MR T1 mapping as a biomarker for liver function in a liver cancer patient cohort. Gaussian decomposition of the k1 image histogram is a feasible method for the creation of patient-specific HF liver masks. The 4*π* FA-SBRT planning guided by k1 maps significantly reduced the mean dose to the HF liver, as well as the volume of HF receiving 6 Gy. This study serves as the groundwork for integrating the 4*π* FA-SBRT strategy into a clinical treatment planning system and supports prospective trials to investigate the efficacy of liver FA treatment.

## Supplementary Material

1

2

Supplementary material associated with this article can be found in the online version at doi:10.1016/j.ijrobp.2025.09.051.

## Figures and Tables

**Table 1:** Patient characteristics

Characteristic	k1 validation cohort	Replanning cohort
Age, mean (range), y	66 (22-91)	66 (41-82)
No. of patients	106	20
Sex, F/M	41/65	5/15
Liver cancer subtypes (HCC/CC + ICC/Mets)	32/17/57	8/5/7
Liver cirrhosis score count (no cirrhosis/CPA/CPB/CPC)	71/27/6/2	7/10/2/1
ALBI score count (grade 1/grade 2/grade 3)	49/51/6	3/14/3
GTV (cc), median (range)	35 (0.31-2500)	67 (3-2500)
Average HF volume (Std) (cm^3^)	903 (346)	908 (285)
Average HF threshold (Std) (%)	45.7 (9.6)	41.4 (9.0)
Prescription range of SBRT, median (Gy)	NA*	(25-50), 43.5

*Abbreviations:* ALBI = albumin-bilirubin; CC = cholangiocarcinoma; CP = Child-Pugh; GTV = gross target volume; HCC = hepatocellular carcinoma; HF = high-function; ICC = intrahepatic cholangiocarcinoma; Mets = metastases; NA = not available; SBRT = stereotactic body radiation therapy; std = standard deviation.

* Not all patients received SBRT.

## Data Availability

The data that support the findings of this study are available on request from the corresponding author.
