# Supplementary material for: Functional Avoidance Liver 4π Stereotactic Body Radiation Therapy Informed by Quantitative Gadoxetic Acid Contrast-Enhanced Magnetic Resonance T1 Mapping
Source: Int J Radiat Oncol Biol Phys. Author manuscript; Available in PMC 2026 Jul 1. (PMC13321400; doi:10.1016/j.ijrobp.2025.09.051)
Supplement: 1 [file NIHMS2183842-supplement-1.docx]

Supplementary material 2

| Replanning Dosimetry Statistics | | | | |
| --- | --- | --- | --- | --- |
| Metric (mean ± σ) | PTV or OAR goals | Coplanar | 4π-without-FA | 4π-with-FA |
| PTV Coverage (%) to receive the prescription | >90% | 6 patients at 90%, 14 at 95% or higher | 6 patients at 90%, 14 at 95% or higher | 6 patients at 90%, 14 at 95% or higher |
| MHFLD* (Gy) | ALARA^ | 9.1 ± 3.2 | 7.8 ± 2.8 | 6.4 ± 2.3 |
| MLD* (Gy) | < 15 | 9.2 ± 3.2 | 7.9 ± 2.9 | 7.0 ± 2.6 |
| Small Bowel (0.5cc point dose, Gy) | < 30 | 21.3 ± 9.7 | 25.4 ± 4.7 | 26.2 ± 3.9 |
| Large Bowel (0.5cc point dose, Gy) | < 32 | 25.8 ± 7.6 | 25.5 ± 7.4 | 25.5 ± 6.9 |
| Stomach (0.5cc point dose, Gy) | < 30 | 24.1 ± 6.0 | 23.44 ± 6.9 | 24.14 ± 7.3 |
| Spinal Canal (0.5cc point dose, Gy) | < 25 | 18.4 ± 4.4 | 14.4 ± 6.6 | 13.2 ± 6.4 |
| *MFLD: Mean High Function Liver Dose, MLD: Mean Liver-PTV Dose.  **^^^**As low as reasonably achievable | | | | |
