# Supplementary material for: Functional Avoidance Liver 4π Stereotactic Body Radiation Therapy Informed by Quantitative Gadoxetic Acid Contrast-Enhanced Magnetic Resonance T1 Mapping
Source: Int J Radiat Oncol Biol Phys. Author manuscript; Available in PMC 2026 Jul 1. (PMC13321400; doi:10.1016/j.ijrobp.2025.09.051)
Supplement: 2 [file NIHMS2183842-supplement-2.pdf]

\\RadOnc_MRI\ABDOMEN\DR. MARY FENG\DR FENG ABDOMEN_2025\RAVE_T1_Rep_25_Pre *
TA: 3:35 min Coil Selection: Auto Voxel Size: 1.4×1.4×5.0 mm³ Rel. SNR: 1.00

Properties

Start measurement without further preparation	On
Wait for User to Start	Off
Start measurements	Single Measurement
Prio Recon	Off
Auto Open Inline Display	Off
Auto Close Inline Display	Off
Load Images to MR View&GO	On
Auto Store Images	On
Disable auto transfer to PACS	Off
Load Images to Stamp Segments	Off
Load Images to Graphic Segments	Off
Graphic segment	Default
Inline Movie	Off

Routine

Slab Group	1
Slabs	1
Distance Factor	20 %
Position	Isocenter
Orientation	Transversal
Phase Encoding Dir.	A >> P
Slices per Slab	48
Slice Oversampling	0.0 %
FOV Read	350 mm
FOV Phase	100.0 %
Slice Thickness	5.00 mm
TR	2.63 ms
TE	1.22 ms
AutoAlign	---

Contrast - Common

TR	2.63 ms
TE	1.22 ms
Magn. Preparation	Non-sel. IR T1map
Flip Angle	5 deg
Fat-Water Contrast	Standard
Contrasts	1
Reconstruction	Magnitude

Contrast - Dynamic

Dynamic Mode	Standard
Measurements	1
Multiple Series	Off

Resolution - Common

FOV Read	350 mm
FOV Phase	100.0 %
Slice Thickness	5.00 mm
Base Resolution	256

Resolution - Common

Slice Resolution	100 %
Trajectory	Radial
Radial Views	1200
Interpolation	Off

Resolution - Acceleration

Slice Partial Fourier	6/8

Resolution - Filter

Raw Filter	Off
Elliptical Filter	Off
Distortion Correction	Off
Normalize	Off
Image Filter	Off

Geometry - Common

Slab Group	1
Slabs	1
Distance Factor	20 %
Position	Isocenter
Orientation	Transversal
Phase Encoding Dir.	A >> P
Slices per Slab	48
Slice Oversampling	0.0 %
FOV Read	350 mm
FOV Phase	100.0 %
Slice Thickness	5.00 mm
TR	2.63 ms
Multi-Slice Mode	Sequential
Series	Ascending

Geometry - AutoAlign

Slab Group	1
Position	Isocenter
Orientation	Transversal
Phase Encoding Dir.	A >> P
AutoAlign	---
Initial Position	Isocenter
L	0.0 mm
P	0.0 mm
H	0.0 mm
Initial Orientation	Transversal
Initial Rotation	0.00 deg

Geometry - Saturation

Special Saturation	None

Geometry - Tim Planning Suite

Set-n-Go Protocol	Off
Table Position	0 mm

Geometry - Tim Planning Suite

Table Position	H
Inline Composing	Off

System - Miscellaneous

Coil Selection	Auto Coil Select
Radial Sorting	Off
MSMA	S - C - T
Sagittal	R >> L
Coronal	A >> P
Transversal	F >> H
Coil Combination	Sum of Squares
Matrix Optimization	Off
Coil Focus	Flat

System - Adjustments

Adjustment Strategy	Standard
B0 Shim	Standard
B1 Shim	TrueForm
CoilShim	Off
Adjustment Tolerance	Auto
Adjust with Body Coil	Off
Confirm Frequency	Never
Assume Silicone	Off

System - Adjust Volume

Position	Isocenter
Orientation	Transversal
Rotation	0.00 deg
A >> P	350 mm
R >> L	350 mm
F >> H	240 mm
Reset	Off

System - pTx

B1 Shim	TrueForm

System - Tx/Rx

Frequency 1H	123.247411 MHz
? Ref. Amplitude 1H	0.000 V
Reset	Off
Image Scaling	1.000

Inline - Subtraction

Subtract	Off
Measurements	1
StdDev	Off
Save Original Images	On

Inline - MIP

MIP Sag	Off
MIP Cor	Off
MIP Tra	Off
MIP Time	Off
Radial MIP	Off

Inline - MIP

Save Original Images	On
MPR Sag	Off
MPR Cor	Off
MPR Tra	Off

Inline - Composing

Inline Composing	Off

Sequence - Part 1

Sequence Name	RAVE
Dimension	3D
Gradient Mode	Fast
Bandwidth	1150 Hz/Px

Sequence - Part 2

Introduction	Off
RF Spoiling	On

Sequence - Special

Parameter Selection	ICE
ICE Program	Radial Regrid NYU
Use MDH angles	On
Gradient calibration	On

Sequence - Assistant

SAR Assistant	Off
